# Quantitative Analysis of a Whole Cardiac Mass Using Dual-Energy Computed Tomography: Comparison with Conventional Computed Tomography and Magnetic Resonance Imaging

**DOI:** 10.1038/s41598-018-33635-0

**Published:** 2018-10-18

**Authors:** Yoo Jin Hong, Jin Hur, Kyunghwa Han, Dong Jin Im, Young Joo Suh, Hye-Jeong Lee, Young Jin Kim, Byoung Wook Choi

**Affiliations:** Department of Radiology and Research Institute of Radiological Science, Yonsei University College of Medicine, Severance Hospital, Seoul, Republic of Korea

## Abstract

It is critical to distinguish between cardiac tumors and thrombi because they require different treatment strategies. Although accurate differentiation of these cardiac masses can be challenging, computed tomography (CT) and magnetic resonance imaging (MRI) are promising tools to improve their diagnosis. This study aimed to assess the diagnostic value of a volume-based quantification strategy using dual-energy CT to differentiate between cardiac tumors and thrombi. We prospectively enrolled 41 patients who had a cardiac mass. All patients underwent electrocardiography gated dual-energy CT. Among them, 28 patients underwent late gadolinium enhancement cardiac MRI. For quantitative analysis, the following parameters of the entire cardiac masses were measured: CT attenuation values in Hounsfield units (HU), iodine concentration (mg/ml), and signal intensity (SI) ratio. A mixed effects model was used to evaluate the significance of differences in mean CT attenuation, mean iodine concentration, and SI ratios between the cardiac tumor and thrombus groups. Diagnostic performance of each parameter was evaluated by constructing a receiver operating characteristics curve. A total of 24 cardiac tumors and 19 cardiac thrombi were analyzed. The mean iodine concentration was significantly higher in tumors than in thrombi (tumors: 2.98 ± 0.23; thrombi: 1.79 ± 0.26, *p* = 0.002). The diagnostic performance of iodine concentration was better than that of post-contrast HU (area under the curve [AUC]: 0.77 vs. 0.51; *p* < 0.001), and worse than that of SI ratio (AUC: 0.89; *p* = 0.04) for differentiation of cardiac tumors and thrombi. Dual-energy CT using volume-based iodine measurements can differentiate between cardiac tumors and thrombi.

## Introduction

Although cardiac masses are not common, they are potentially high-risk sources of embolism, so accurate and prompt diagnosis is important^1^. It is also clinically important to differentiate cardiac tumors from cardiac thrombi, as they are managed differently^2,3^.

Cardiac magnetic resonance imaging (MRI) has become a mainstream imaging modality for differential diagnosis of cardiac tumors because it provides an unrestricted field of view and superior tissue characterization without radiation exposure^4,5^. A previous study demonstrated that late gadolinium enhancement (LGE) cardiac MRI is a useful non-invasive modality to differentiate a cardiac tumor from a thrombus^6^.

Multi-detector computed tomography (CT) is increasingly being used for cardiac imaging^7^. However, CT lacks the superior contrast abilities of MRI and is therefore not suitable for differentiation between cardiac tumors and thrombi^6,8^.

Dual-energy CT using rapid kilovoltage (kVp) switching allows simultaneous acquisition of low and high kVp datasets. It is possible to differentiate iodine from other materials using the material decomposition method^9^. This is valuable for differentiating between iodine-enhancing lesions such as cardiac tumors, and non-enhancing lesions such as thrombi. A previous study demonstrated that dual-energy CT was useful for characterizing cardiac masses and differentiating between cardiac tumors and thrombi based on quantification of the iodine content of the mass^8^. However, the lack of electrocardiography synchronization in dual-energy mode and evaluation of only certain areas of the mass were limitations of the study.

The recent development of simultaneous electrocardiography synchronization in rapid kVp-switching dual-energy mode yields good quality CT images without artifacts or image degradation. In addition, newly developed software allows whole tumor segmentation, which enables whole-tumor evaluation.

The purpose of this study was to assess the diagnostic value of a volume-based quantification using dual-energy cardiac CT to differentiate between cardiac tumors and thrombi, and to compare quantitative CT values with LGE parameters.

## Results

### Demographic and baseline characteristics of patients

The demographic data and baseline clinical characteristics of the 41 patients are summarized in Table 1. The mean diameter of cardiac tumors was significantly larger than that of thrombi (*p* < 0.001, all longest and shortest diameters). Patients with thrombi had higher incidences of smoking, history of cerebrovascular disease, heart disease, and arrhythmia than those with cardiac tumors. Other clinical characteristics of the two groups were not significantly different. A total of 24 cardiac tumors and 19 cardiac thrombi were identified among the 41 patients, as two patients each had two thrombi. Among the 24 cardiac tumors, 15 were benign, and included myxoma (n = 9), cavernous hemangioma (n = 2), lipoma (n = 2), angiofibroma (n = 1), and papillary fibroelastoma (n = 1). Nine were malignant and included lymphoma (n = 4) and metastasis (n = 5). All tumors were confirmed by surgical excision or biopsy. One thrombus was confirmed by surgical excision; all other thrombi disappeared after anticoagulation therapy, which was considered proof of diagnosis.

**Table 1:** Demographic and baseline characteristics of patients.

	Cardiac tumors (n = 24)	Cardiac thrombi (n = 17)*	*P* value
Male	10 (58.82)	8 (57.14)	0.786
Age (years)	58.13 ± 17.46	59.47 ± 12.64	0.788
Axis size (mm)			
Long	39.48 ± 14.86	23.46 ± 12.81	0.001
Short	29.03 ± 10.99	15.13 ± 7.69	< 0.001
Location			0.133
Left atrium	7 (29.17)	11 (57.89)	
Left ventricle (LV)	3 (12.50)	4 (21.05)	
Right atrium	10 (41.67)	4 (21.05)	
Right ventricle	4 (16.67)	0 (0.00)	
Diabetes mellitus	5 (20.83)	6 (35.29)	0.476
Hypertension	8 (33.33)	9 (52.94)	0.209
Smoking	5 (20.83)	9 (52.94)	0.033
Hyperlipidemia	1 (4.17)	2 (11.76)	0.560
Prior CVA/TIA	2 (8.33)	8 (47.06)	0.008
Heart disease^†^	1 (4.17)	8 (47.06)	0.002
Arrhythmia	6 (25.00)	10 (58.82)	0.029

Note: Values are given as mean ± SD, Data in parentheses are percentages.

^*^As two patients each had two thrombi, 17 patients were included in the demographics and 19 lesions were included in the location analysis.

^†^Heart disease includes valvular heart disease and myocardial infarction.

CVA, cerebrovascular accident; TIA, transient ischemic attack.

### Quantitative analysis of dual-energy cardiac CT and MRI of cardiac tumors and thrombi

All 41 patients underwent dual-energy CT; 28 of the 41 patients underwent cardiac MRI. There were no adverse events during CT or MRI examinations. Among the 24 cases of cardiac tumors, 16 had available MRI data; among 19 cases of cardiac thrombi, 12 had available MRI data within a 2-week timeframe.

**Figure 1:**
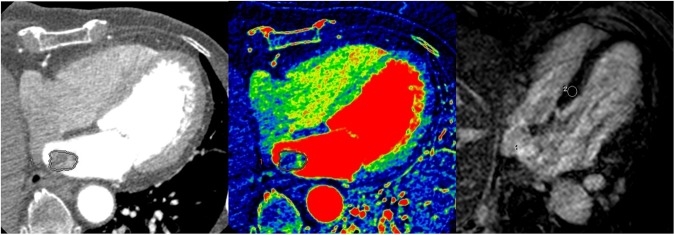
A 72-year-old female patient diagnosed with a myxoma. (**A**) On enhanced CT, a 2.4 × 1.5-cm well-defined mass was noted in the left atrium. The volume of the mass was 3.37 cm^3^ and the CT attenuation value of the intra-cardiac mass was 40.00 HU. (**B**) On the iodine (water) image, the iodine concentration of the mass within the VOI was 5.04 mg/ml. (**C**) On LGE image, the mass shows strong contrast enhancement; the SI ratio of the mass was 20.50.

**Figure 2:**
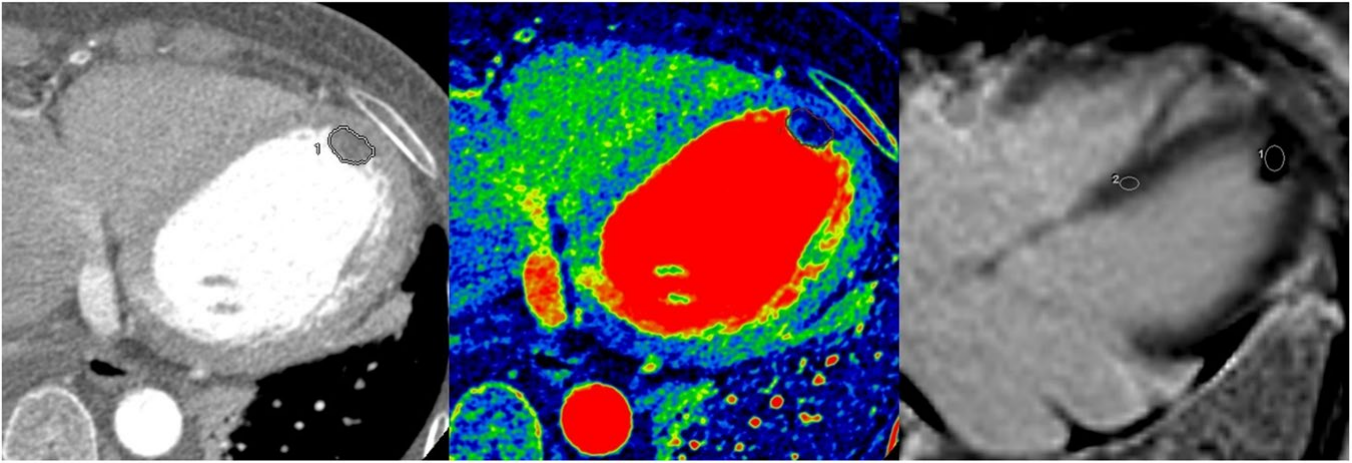
A 53-year-old female patient diagnosed with a thrombus. (**A**) On enhanced CT, a 1.5 × 1.0-cm well-defined mass was noted in the apex of left ventricle. The volume of the mass was 0.81 cm^3^ and the CT attenuation value of the intra-cardiac mass was 59.0 HU. (**B**) On the iodine (water) image, the iodine concentration within the VOI was 1.05 mg/ml. (**C**) On LGE image, the mass shows dark signal intensity without enhancement; the SI ratio of the mass was 0.89.

**Figure 3:**
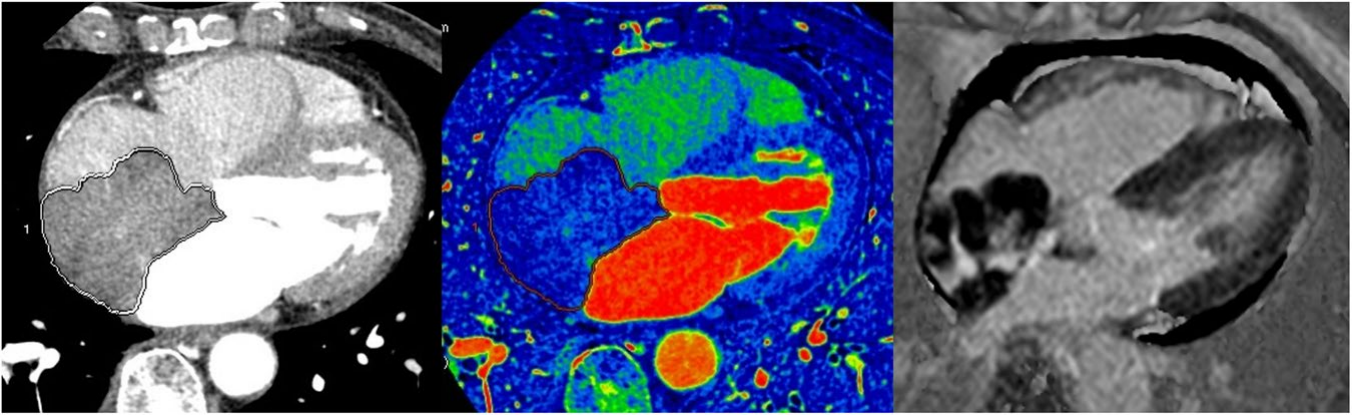
A 72-year-old female patient diagnosed with a lymphoma. (**A**) On enhanced CT, a 6.8 × 4.8-cm lobulated mass was observed in the right atrium. The volume of the mass was 47.1 cm^3^ and the CT attenuation value of the intra-cardiac mass was 70.0 HU. (**B**) On the iodine (water) image, the iodine concentration within the VOI was 1.67 mg/ml. (**C**) MRI was performed three days later. On the LGE image, the mass shows central enhancement, the SI ratio of the mass was 4.16. Minimal pericardial effusion is noted.

The detailed quantitative values for the cardiac tumors and thrombi are shown in Table 2. The volume of the cardiac tumors was significantly larger than that of the thrombi (24.52 ± 3.76 vs. 3.98 ± 4.23 -> 4.22, p = 0.001 -> p < 0.001). The mean CT attenuation values of the cardiac tumors and thrombi measured on post-contrast CT images were not significantly different (73.03 ± 8.78 vs. 74.50 ± 9.87 Hounsfield units (HU), respectively; *p* = 0.912). On iodine maps, the mean iodine concentration (mg/mL) was significantly higher in the cardiac tumors than in the thrombi (cardiac tumors: 2.98 ± 0.23; cardiac thrombi: 1.79 ± 0.26; *p* = 0.002). On MRI, the signal intensity (SI) ratio was also significantly different between the cardiac tumors and thrombi (6.90 ± 1.25 -> 0.12 vs. 1.43 ± 1.44 -> 0.14 respectively; *p* = 0.008). Representative cases of the cardiac tumors and thrombi are shown in Figs 1–3.

**Table 2:** Quantitative analysis of dual-energy CT and MRI findings for cardiac tumors and thrombi.

	Cardiac tumors (n = 24)					Cardiac thrombi (n = 19)	*P* value (tumors vs. thrombi)
	Lymphoma (n = 4)	Metastasis (n = 5)	Myxoma (n = 9)	Other* (n = 6)	All (n = 24)		
Volume (cm^3^)	32.14 ± 8.77	36.50 ± 7.84	24.24 ± 5.84	9.89 ± 7.16	24.52 ± 3.76	3.98 ± 4.22	<0.001
CT attenuation value (HU)	94.63 ± 21.11	88.60 ± 18.88	74.11 ± 14.08	44.02 ± 17.24	73.03 ± 8.78	74.50 ± 9.87	0.912
Iodine concentration (mg/mL)	3.04 ± 0.58	2.96 ± 0.52	3.33 ± 0.39	2.43 ± 0.47	2.98 ± 0.23	1.79 ± 0.26	0.002
SI ratio^†^	4.13 ± 2.76	3.31 ± 3.39	10.30 ± 1.95	5.92 ± 2.14	6.90 ± 0.12	1.43 ± 0.14	0.008

Note: Estimated mean ± SE (standard error) by the mixed effects model.

*Other tumor included cavernous hemangioma (n = 2), lipoma (n = 2), angiofibroma (n = 1), and papillary fibroelastoma (n = 1).

^†^For SI ratio, sample size was 16 for cardiac tumors and 12 for cardiac thrombi.

HU, Hounsfield units; SI, signal intensity.

#### Subgroup analysis of the quantitative results

Differences in quantitative values were analyzed between benign (n = 15: myxoma [n = 9], cavernous hemangioma [n = 2], lipoma [n = 2], angiofibroma [n = 1]) and malignant tumors (n = 9: lymphoma [n = 4], metastasis [n = 5]). However, there was no significant difference in parameters between the benign and malignant tumors, including HU (69.02 ± 7.21 vs. 91.28 ± 14.00, *p* = 0.17), iodine concentration (2.31 ± 0.22 vs. 2.99 ± 0.42, *p* = 0.16), or SI ratio (4.72 ± 1.19 vs. 3.80 ± 2.56, *p* = 0.75). According to the tumor type, there were significant differences in the iodine concentration between myxomas and thrombi (3.33 ± 0.39 vs. 1.79 ± 0.26, *p* = 0.002) and metastasis and thrombi (2.96 ± 0.52 vs 1.79 ± 0.26, *p* = 0.042). The SI ratio was significantly different between myxoma and thrombi (10.30 ± 1.95 vs 1.43 ± 0.14, *p* = 0.001) and other benign tumors and thrombi (5.92 ± 2.14 vs 1.43 ± 0.14, *p* = 0.026). However, there was no significant difference in iodine concentration or the SI ratio between other tumor subtypes. HU showed no significant difference between any tumor subtype.

### Diagnostic performance of dual-energy CT and MRI

To evaluate the diagnostic performance of dual-energy CT and MRI for differentiation of cardiac tumors from thrombi, the receiver operating characteristics (ROC) curve and the area under the curve (AUC) of each parameter were evaluated.

When iodine concentration was used to differentiate cardiac tumors from thrombi, the AUC was 0.77 (95% confidence interval [CI]: 0.63–0.91). The optimal cutoff value of iodine concentration for differentiation between tumor and thrombus was 2.55 mg/mL; sensitivity and specificity were 66.70% and 79.00%, respectively.

The AUC of post-contrast HU was 0.51 (95% CI: 0.34–0.69). There was a significant difference between the AUC of iodine concentration and post-contrast HU (difference in AUC: −0.38 [95% CI: −0.49–−0.27]; *p* < 0.001).

**Figure 4:**
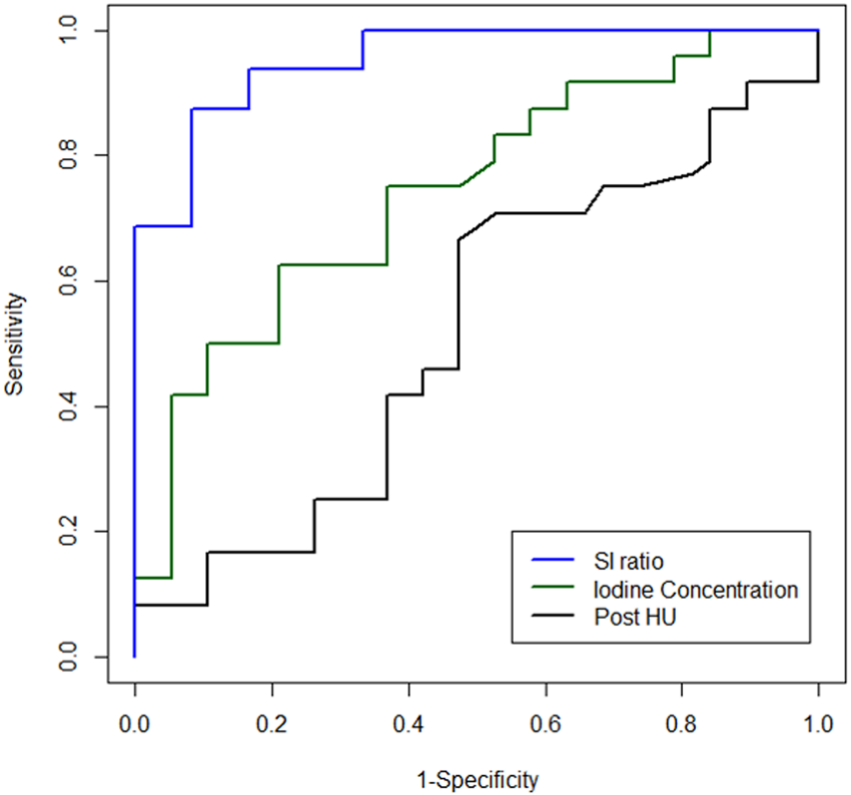
ROC curves of post-contrast HU, iodine concentration, and SI ratios. The AUC of iodine concentration was higher than that of post-contrast value in HU (AUC; 0.51 vs. 0.77; *p* < 0.001), and lower than of the SI ratio (AUC; 0.77 vs. 0.89; *p* = 0.04).

The AUC of the SI ratio was 0.89 (95% CI: 0.75–1.00), which was significantly higher than that of the iodine concentration (difference of AUC: −0.13 [95% CI: −0.26–−0.004]; *p* = 0.043). The ROC curves of post-contrast HU, iodine concentration, and SI ratio are included in Fig. 4.

### Correlation between dual-energy CT and MRI data

The correlation between the SI ratio and iodine concentration was good (0.74 [95% CI: 0.51–0.85]) in all patients; the correlation between the SI ratio and post-contrast CT attenuation value was relatively low (0.41 [95% CI: −0.24–0.87], Table 3).

**Table 3:** Correlation between SI ratio and each variable using replicated measurements (95% CI).

Parameter	All patients	Cardiac tumors (n = 24)	Cardiac thrombi (n = 19)
Iodine concentration (mg/ml)	0.74 (0.51, 0.85)	0.77 (0.50, 0.92)	0.39 (−0.03, 0.98)
CT attenuation value (HU)	0.41 (−0.24, 0.87)	0.43 (−0.46, 0.96)	0.55 (0.12, 0.98)

SI, signal intensity; CI, confidence interval; HU, Hounsfield units.

There was excellent inter-observer agreement between the two radiologists regarding the measurement parameters of dual-energy CT and MRI. The intra-class correlation coefficients (ICC) for iodine concentration, SI ratio, and post-contrast HU were 0.98 (95% CI: 0.97–0.99), 0.97 (95% CI: 0.93–0.98), and 0.98 (95% CI: 0.97–0.99), respectively. Radiation exposure was estimated from the dose-length product (DLP). The calculated mean radiation dose was 4.58 mSv (DLP: 146.0–462.2 mGy × cm).

## Discussion

This study aimed to evaluate the diagnostic performance of dual-energy CT and compare it with those of LGE and CT parameters for the differentiation of cardiac tumors and thrombi. We demonstrated that volume-based iodine measurements using dual-energy CT provide a feasible quantitative parameter for differentiating cardiac tumors from thrombi.

Cardiac MRI plays an important role in the assessment of cardiac masses. It allows multi-parametric characterization of cardiac masses using topography, morphology, SI, or pattern of enhancement of cardiac tumors^3,10,11^. However, MRI has several disadvantages, including a long scanning time, and it cannot be used to examine patients with implanted metallic devices.

LGE imaging, widely used to distinguish between viable and infarcted myocardium, allows identification of a thrombus in the absence of contrast enhancement. Several studies have demonstrated that LGE can accurately delineate a cardiac thrombus from the myocardium and chamber cavity based on tissue characteristics, irrespective of location or morphology^12–15^. Previous studies have demonstrated that the contrast enhancement pattern obtained from LGE can be used to differentiate between cardiac tumors and thrombi^12–14^. In an earlier study, cardiac tumors presented as high-SI masses compared to viable myocardium, reflecting gadolinium contrast enhancement. In comparison, thrombi presented as low-SI masses (either homogeneously black, or as a black border around a central gray zone), indicating avascular tissue^6^. The present study also demonstrated that LGE imaging can distinguish various cardiac tumors from thrombi, which showed higher diagnostic value than CT parameters.

CT is a widely available and highly reproducible imaging technique that can quickly, accurately, and noninvasively determine cardiac anatomy. CT has several advantages over MRI, including coverage of the whole myocardium in a short scanning time, widespread availability, and simultaneous evaluation of coronary arteries. Additionally, CT can replace MRI in cases with contraindications. Recently, dual-energy CT was proved to be a non-invasive modality that allows differentiation of cardiac myxomas from thrombi^8^. A previous study revealed that iodine concentration measurements were significantly higher in tumors than in thrombi, while mean CT attenuation values were not significantly different between the two mass types^8^. However, the limitation of that study was that only myxomas were evaluated. In addition, iodine quantification in the previous study was limited by the lack of synchronization of electrocardiography with cardiac movements using dual-energy mode. Therefore, identification of a simple quantitative parameter to differentiate cardiac tumors and thrombi within a heterogeneous cardiac mass is potentially challenging.

We conducted this study to investigate the ability of dual-energy CT to differentiate between cardiac tumors and thrombi. Iodine quantification may have measurement variability for technical (i.e., scanner, acquisition technique, patient compliance) and biological (variability of tumor blood supply) reasons^16,17^. In this study, we quantified the whole volume of the cardiac mass using electrocardiographic synchronization with cardiac movements using dual-energy mode.

According to our results, mean CT attenuation values were not significantly different between cardiac tumors and thrombi, while mean iodine concentration was significantly higher in cardiac tumors than in thrombi. In addition, the diagnostic performance of iodine concentration was significantly higher than that of post-contrast HU measurements for distinguishing cardiac tumors from thrombi. However, the AUC of iodine concentration was significantly lower than that of the SI ratio of LGE imaging. This result suggests that dual-energy CT is more useful for differentiation between tumors and thrombi than conventional CT. Dual-energy CT is an accurate approach for defining whether a cardiac mass is enhanced. Iodine quantification appears more accurate than standard enhancement measurements when the volume of interest (VOI) covers the entire mass. Although cardiac MRI is a reference modality for evaluation of cardiac masses^10,11^, dual-energy CT can play a complementary role to that of MRI in cases when MRI is not available or in which echocardiography or conventional CT results are inconclusive.

Several limitations of this study should be acknowledged. First, a small sample size and limited histologic types of cardiac masses were included. As cardiac tumors are a rare disease entity, it was difficult to enroll a large population for this prospective study. However, according to our sample size estimation, 19 subjects in each group could have a statistical power of 0.8. Second, our contrast injection protocol may have significantly influenced the results. Few studies have investigated the role of dual-energy CT in cardiac tumor imaging and, to our knowledge, there are neither data on contrast media administration nor standard scanning protocols available. Previous studies have found that dual-energy CT performed using a double injection protocol showed both high sensitivity and high specificity for detection and differentiation of cardiac thrombi and blood stasis^18^. As most thrombi do not show enhancement (whereas tumors often do), a double injection protocol was used to achieve a sufficient attenuation difference between iodine-enhancing and non-enhancing lesions. Third, cardiac MRI and CT were not performed on the same day; the median time interval between the two techniques was 4.8 days. In addition, only 28 of the 41 patients underwent MRI.

In conclusion, dual-energy CT using volume-based iodine measurements can be used to differentiate between cardiac tumors and thrombi. Although dual-energy CT is not always performed in routine practice due to the radiation exposure associated with this technique, it can be a helpful complementary tool to MRI in cases when MRI is not available or in which echocardiography or conventional CT provide inconclusive results.

## Materials and Methods

### Patient selection

This single-center prospective observational study was approved by the institutional review board of Yonsei University Health System (approval number: 4-2012-0396). Informed consent was obtained from all patients. All studies were performed in accordance with the relevant guidelines and regulations.

**Figure 5:**
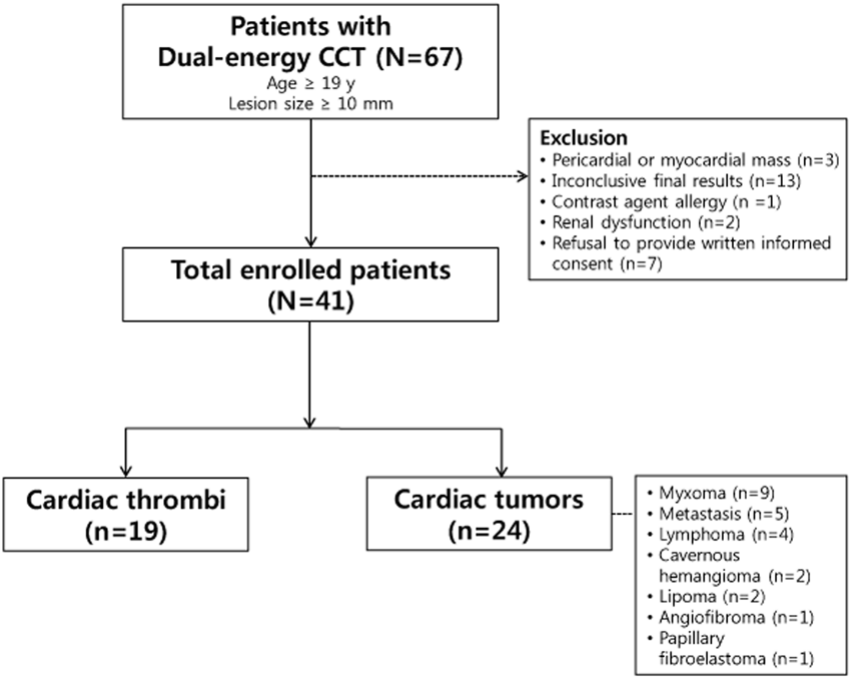
A flow diagram of the patient selection process.

From December 2012 to November 2016, 67 consecutive patients with a suspicious intracardiac mass on echocardiography or conventional CT were prospectively enrolled to undergo dual-energy CT and MRI. Patients met inclusion criteria if they were more than 19 years of age and had a cardiac mass lesion, which was defined as a lesion greater than 10 mm in the longest diameter. Exclusion criteria included: pericardial or myocardial mass (n = 3), inconclusive final results (n = 13), contrast agent allergy (n = 1), renal dysfunction (n = 2), and refusal to provide written informed consent (n = 7). Finally, 41 patients (25 men, 16 women; mean age, 59.30 years; range 19–87 years) were included. The patient selection process is summarized in Fig. 5.

Final diagnoses were based on either pathological results from surgery or biopsy, a follow-up examination including echocardiography, or MRI after anticoagulant treatment without additional treatment.

### Dual-energy cardiac computed tomography (CT) examination

Dual-energy CT scans were performed with a 64-row multidetector CT scanner (Discovery CT750 HD; GE Healthcare, Chicago, IL, USA). No beta-blockers were used to regulate heart rate. The mean heart rate was 64.5 ± 9 beats per min (range 47–90 bpm) during the CT examination.

For contrast injection, we followed a double injection method that requires two injections and one scan^18,19^. For the first bolus injection, 50 mL of the nonionic contrast agent iopamidol (370 mg of iodine/mL, Iopamiro^®^, Bracco Imaging, S.p.A., Milan, Italy), was administered via power injector (Envision CT, Medrad, Warrendale, PA, USA) at 5 mL/s into the right antecubital vein. A region of interest (ROI) was plotted inside the ascending aorta and a bolus geometry curve was acquired. Curve diagrams were analyzed to determine the optimal scan delay.

The second bolus injection of 70 mL of contrast agent was administered intravenously at the same rate 180 s after the end of the first bolus injection, followed by 50 mL of contrast-saline mixture at a ratio of 30:70. The scan was begun after the optimal scan delay using the gemstone spectral imaging (GSI) mode (electrocardiography-gated dual-energy CT mode). The scan parameters were: detector collimation = 64 × 0.625 mm; gantry rotation time = 0.5 s; tube voltage = 140/80 kV; tube current = 630 mA; pitch = 1.375:1. All images were reconstructed with a 0.625-mm slice thickness.

### Image analysis of dual-energy CT

Dual-energy CT data were assessed by two radiologists with 8 and 11 years of cardiac imaging experience. These radiologists were blinded to patient data. All scan images were transferred and computed in GSI Volume Viewer mode on a dedicated workstation equipped with dual-energy post-processing software (GE workstation, Volumeshare 4.4.5, GE Healthcare).

Size (longest diameters of the long and short axes images) and volume of the intra-cardiac masses were measured. The two radiologists performed these measurements independently and inter***-***observer reliability was calculated. The cardiac mass locations were also recorded. Locations were classified as follows: left atrium, left ventricle, right atrium, or right ventricle.

After processing in GSI volume viewer mode, three modes of images, namely monochromatic 70-keV images (which represent conventional 120-kV post-contrast CT images), material-suppressed iodine (MSI) images (which represent conventional 120-kV pre-contrast CT images), and iodine (water) images were displayed on the workstation, side-by-side, to show the same level of the mass.

**Figure 6:**
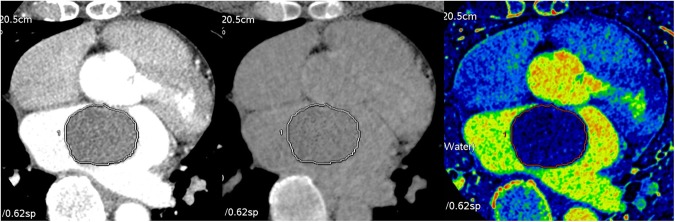
A representative image set for quantitative analysis of dual-energy CT of a cardiac tumor. Three modes of images were obtained using dual-energy CT: (**A**) a monochromatic 70 keV image representing the post-contrast CT image, (**B**) a MSI image representing the pre-contrast CT image, and (**C**) an iodine (water) image; A volume of interest covering the entire cardiac mass was drawn on the image of each mode on the identical level of the cardiac tumor.

For quantitative analysis, the entire VOI of the mass was isolated using semi-automatic segmentation. If the segmented border of the mass was incorrect, the reviewers manually corrected the VOI per the border of the mass on slices that included the cardiac mass on axial monochromatic 70-keV images. After drawing a VOI covering the entire mass, the segmented VOI of the mass was propagated to the other two modes’ alternate images to cover the same VOI of the tumor across the three images **(**Fig. 6**)**.

The mean CT attenuation densities of the entire cardiac masses in post-contrast CT images (monochromatic 70-keV images) and pre-contrast CT images (MSI images) were measured. In iodine (water) images, the mean iodine concentration (mg/ml) for the same VOI covering the entire tumor was also measured.

### Cardiac MRI examination

MRI and dual-energy CT were performed within a 2-week timeframe. MRI was performed with a 1.5 Tesla (T) MR unit (Gyroscan Intera, Philips Medical Systems, Amsterdam, The Netherlands). LGE was performed using a segmented inversion recovery radiofrequency spoiled gradient echo (T1-TFE) sequence (TR/TE = 5.3/1.6 ms, flip angle = 15°, slice thickness = 10 mm, field of view = 360 mm, matrix = 512 × 512, number of signal average = 2), 10 min after intravenous injection of gadolinium-DTPA at a dose of 0.2 mmol/kg. The inversion time was determined using a dedicated TI-determining sequence (Look-Locker); the range was 220–300 ms.

### MRI analysis

The same radiologists who performed the CT image analysis reviewed the LGE data independently using the same workstations and were again blinded to patient data; inter***-***observer reliability was calculated. For the quantitative analysis, the two reviewers measured the SI in the operator-defined ROI in the normal myocardium and cardiac masses on the LGE (magnitude) images; mean SI values were used in the analyses. For the cardiac masses, the ROI was drawn to cover as much of the lesion as possible on one representative image. The SI ratios of normal myocardium and cardiac masses were calculated.

### Sample size consideration

From previous study data^8^, the expected AUC of the iodine concentration and post-contrast HU were 0.8 and 0.6, respectively. The correlation between the two measurements for each group was positively set at 0.6. A statistical power of 0.80 and type I error of 0.05 required 19 subjects per group. The sample size estimation was performed using PASS software (version 12, NCSS, LLC., Kaysville, UT, USA)^20^.

### Statistical analysis

Categorical demographic data are expressed as numbers and percentages and were compared between the cardiac tumor and thrombus groups using the chi-square or Fisher’s exact tests. Continuous variables were expressed as means ± standard deviations. The error of mean is defined as one standard deviation. They were analyzed using a Shapiro-Wilk test to confirm a normal distribution and compared with a Student’s *t-*test. Differences in volume, mean post-contrast HU, mean iodine concentration, and SI ratio between the groups (cardiac tumor vs. thrombus, benign vs. malignant tumor, subtypes of tumor) were evaluated with a mixed effects model. The two groups were regarded as fixed effects, patients were considered a random effect. Correlation coefficients with 95% CI between the SI ratio and each variable were estimated based on a linear mixed model to account for replicated measurements of the two readers^21^. Diagnostic performance of each parameter was evaluated by an ROC curve with multi-reader and multi-case analysis using the statistical software iMRMC version 2.7 (Food and Drug Administration Center for Devices and Radiological Health, Silver Spring, MD, USA)^22^. The significance of differences between the AUCs of mean post-contrast HU and iodine concentration was evaluated using Delong’s method^23^; a different statistical method proposed by Zhou and Gatsonis^24^ was used to include partially paired CT data in AUC comparisons between the iodine concentration and SI ratio. We also determined the optimal cut-off value of iodine concentration to differentiate between cardiac tumors and thrombi using Youden’s index to yield the maximal sum of the sensitivity and specificity^25^. ICCs were used to evaluate inter-observer agreement for measurements of parameters using a two-way random effects model. Patients and radiologists were considered to be random effects. *P* values < 0.05 were considered significant. All statistical analyses were performed using IBM SPSS Statistics for Windows (Version 21.0, IBM Corp., Armonk, NY, USA).

## Data Availability

All data generated or analyzed during this study are included in this published article.
